# SWAT 116: the effectiveness of an infographic to improve trial recruitment—a randomised study within a trial (SWAT)

**DOI:** 10.1186/s13063-026-09830-0

**Published:** 2026-07-07

**Authors:** C. E. Arundel, F. Wiggins, K. Baird, C. Fairhurst, A. Mott, S. Swan, J. Wilkinson, S. Zahra, I. Chetter

**Affiliations:** 1 York Trials Unit, Department of Health Sciences, Faculty of Science, University of York, Lower Ground Floor ARRC Building, York, YO10 5DD UK; 2 University of Hull, Hull, HU6 7RX UK; 3 Hull York Medical School, Hull, HU6 7RX UK; 4 Hull University Teaching Hospitals NHS Trust, Hull, HU3 2JZ UK

**Keywords:** SWAT, Study within a trial, Retention methods, Embedded randomised controlled trial

## Abstract

**Background:**

Randomised controlled trial recruitment is challenging. Recruitment and informed consent processes require provision of a participant information sheet (PIS). Visual presentation of information, in the form of an infographic, is increasingly utilised, despite limited effectiveness evidence. This Study Within A Trial (SWAT) assessed if a PIS plus an infographic increases recruitment relative to PIS alone.

**Methods:**

A two-arm cluster randomised SWAT (1:1 allocation ratio at the site level), embedded within the SWHSI-2 trial. The primary outcome was recruitment rate analysed via mixed effects logistic regression using minimisation factors as fixed effects and site as a random effect. Secondary outcomes were the proportion of participants screened but not recruited, retention rate, and cost.

**Results:**

All 29 SWHSI-2 sites were randomised to the SWAT (14 intervention; 15 control). Following SWAT implementation, 1634 patients were screened, with 1068 patients eligible (PIS plus infographic, 662/1008 [65.7%]; PIS only, 406/626 [64.9%]) and 585 randomised into the SWHSI-2 trial (PIS plus infographic, 342/662 [51.7%]; PIS only, 243/406 [59.9%]; (OR 0.61, 95% CI 0.23 to 1.62, *p* = 0.32). More patients were screened but not randomised in the intervention group compared to the control group (*n* = 666, 66.1% vs *n* = 383, 61.2%). Meta-analysis of two studies found the infographic intervention reduced participant recruitment (OR = 0.61, 95% CI 0.31 to 1.23).

**Conclusion:**

Two SWAT replications have found that including an infographic alongside a PIS does not increase participant recruitment, but estimates were imprecise. Replications in other populations and exploration of infographic content and design remain warranted.

**Supplementary Information:**

The online version contains supplementary material available at https://doi.org/10.1186/s13063-026-09830-0.

## Background

The challenges to randomised controlled trial (RCT) recruitment have been well documented, with approximately 50% of RCTs failing to recruit to target [1–4]. This leads to research waste, by virtue of increased costs and reduced statistical power [5, 6]. Effective interventions to improve recruitment of participants to RCTs are therefore required.

A Study Within A Trial (SWAT)—an evaluation nested within a larger host trial—allows development and assessment of such interventions [7]. SWATs are often randomised which ensures the causality of intervention effectiveness can be assessed [8]. Given that SWATs are embedded in host trials, they cannot often be purposefully powered or consequently provide definitive evidence alone, but they aim to meaningfully contribute to the evidence base and be combined in meta-analyses with other studies to identify effective and ineffective interventions [9].

Participant information sheets (PIS) are a required information provision as part of the recruitment and informed consent process for a RCT [10]. As reported in the Cochrane review of recruitment strategies for RCTs, 35 studies have assessed modification of information given to participants for trial recruitment, of which 17 (49%) were deemed to be at high risk of bias [11]. Of the remaining studies only three (17%) had high GRADE certainty, of which only one related to information provision; tailored user tested information sheets did not make much difference to recruitment (risk difference 1%; 95% CI −1% to 3%) [11].

Patient information sheets are routinely provided as paper-based documentation. Variability of patient’s health literacy can mean that some individuals struggle to comprehend this, often extensive, document and this may deter individuals from participating research studies [12].

This is a significant issue, given that informed consent is an integral part of the ethical conduct of a trial [13]. Improving information provision to better facilitate comprehension is therefore necessary. Previous research has suggested that modifications to the PIS (e.g. layout, use of colour and images) are both preferred by patients and may also increase the number of participants attending a screening visit [14]. Infographics that provide key information in an engaging format via a combination of text, images, and data visualisation are therefore increasingly used by researchers and research funders to present an overview of their work [15, 16]. Previous investigation of the effectiveness of infographics for improving patient knowledge has provided mixed results [17, 18]; however, one study did identify that infographics improved reader experience and user-friendliness [19]. The use of infographics as a recruitment tool may help to improve patient understanding of a trial by addressing challenges with regard to health literacy or language barriers [20]. Improving one or more of these is posited to have an impact on recruitment and retention rates [21].

The James Lind priority setting for recruitment to RCTs (PRioRiTy I) has identified 20 priorities for researchers evaluating methods of RCT recruitment [22]. The use of an infographic alongside standard participant information fits with the PRioRiTy I recruitment question number 4; What are the best approaches for designing and delivering information to members of the public who are invited to take part in a RCT [22]. There is therefore a need to test the impact of an infographic in the context of trial recruitment, for which there is currently limited evidence. A single SWAT has thus far been conducted, which found that a higher proportion of participants were recruited if they were allocated to not receive an infographic (OR 0.62, 95% CI 0.23 to 1.66, *p* = 0.34); however, uncertainty is present around the estimate [21, 23]. This paper reports on another infographic SWAT, and at least two further replications of an infographic SWAT are ongoing currently which will add to the evidence base and hopefully, when combined in a meta-analysis, provide a more conclusive, precise, and reliable effect estimate [24, 25].

The objective of this SWAT was to answer the following question: Does giving potential participants an infographic in addition to the trial PIS increase recruitment to the Surgical Wounds Healing by Secondary Intention 2 (SWHSI-2) trial relative to providing the PIS alone?

## Methods

### Design

A two-arm, parallel group, cluster randomised controlled SWAT was undertaken within the SWHSI-2 trial. Recruitment sites were randomised 1:1 to provide an infographic in addition to the PIS when approaching prospective study participants (intervention) or to just use the PIS (control).

The SWAT protocol (number 116) can be found at: https://www.qub.ac.uk/sites/TheNorthernIrelandNetworkforTrialsMethodologyResearch/FileStore/Filetoupload959361en.pdf

The SWHSI-2 trial protocol and results have been previously published [26, 27]. In brief, the SWHSI-2 trial was a pragmatic, multicentre, randomised (1:1)-controlled trial to assess the clinical and cost-effectiveness of negative-pressure wound therapy (NPWT) versus usual care for surgical wounds healing by secondary intention (SWHSI). The primary outcome was time to wound healing (defined as complete epithelial cover with no scab in situ) in days from randomisation. Ultimately the findings do not support the use of NPWT to augment SWHSI healing [27].

The SWAT was considered low risk as the information provided in the infographic was supplementary to the PIS, and so all participants received the same information irrespective of SWAT allocation. Participants were not therefore informed of their participation in the SWAT and so did not provide informed consent for their involvement [7]. This is commonplace in low-risk SWATs such as these, particularly in the context of a recruitment SWAT where providing information on the SWAT and/or obtaining consent can be confusing to potential participants given they have not yet been approached about the host trial [28]. Furthermore, provision of information and/or consent has potential to jeopardise the SWAT through the Hawthorne effect and/or resentful demoralisation [28].

The SWAT was approved by the Research Ethics Committee Yorkshire and Humber–Leeds East (19/YH/0054).

### Participants

The SWAT was embedded in the SWHSI-2 trial on 09.03.2020; therefore, all participants approached for recruitment on or after this date were eligible for inclusion in the SWAT.

SWAT data were collected in the 29 participating NHS hospitals and community NHS trusts via screening and recruitment data routinely collected for the SWHSI-2 trial.

### Intervention

The SWAT intervention was an infographic (see Supplementary File 1) provided in addition to a PIS (see Supplementary File 2) at the point of recruitment. The control group received just the approved SWHSI-2 PIS.

### Outcomes

#### Primary outcome

- Difference in recruitment rate between sites allocated to use the infographic and those allocated to use the PIS alone. Recruitment rate was defined as the proportion of participants in each SWAT group randomised into the host trial and was calculated as the total number of participants recruited in each arm, divided by the total number of participants randomised to each SWAT arm who were eligible to be randomised to the host trial.

#### Secondary outcome

- Difference in proportion of participants screened but not randomised into the host trial. This was calculated as the total number of potential participants found to be ineligible, unwilling to consent or not randomised due to any other reason divided by total number of participants screened in each SWAT group.
- Difference in retention rate of randomised participants. Retention rate was defined as the proportion of participants in each SWAT group who returned a follow-up form and was calculated as the total number of participants followed up in each arm, divided by the total number of participants randomised to each SWHSI-2 trial arm.
- Cost per participant recruited.

No changes were made to the SWAT outcomes once the SWAT started.

### Sample size

The SWAT sample size was dependent on the number of recruitment sites set up in the host SWHSI-2 trial, and the number of participants these sites approached for participation [26]; therefore, no formal sample size calculation was performed, which is in line with general SWAT methodology [7, 29]. As the SWAT was implemented part way through the SWHSI-2 trial, only potential participants approached on or after 09.03.2020 were included in the analysis. 

### Randomisation

Recruitment sites were randomised 1:1 using minimisation on the following factors: (i) whether the site was proposing to recruit cross specialty or in a single specialty; and (ii) expected number of eligible participants per month as reported on the site feasibility assessment, cut at the median.

Allocation concealment was achieved through the generation of the allocation sequence, as sites commenced study set up, by the SWHSI-2 trial statistician based at York Trials Unit, University of York, and who was independent to the trial team.

The randomised allocations were then provided to the central coordinating team for implementation. The statistician was not involved in preparing or sending the infographics to sites.

### Blinding

It was not possible to blind either the central coordinating team or the recruiting site staff to the SWAT allocation. Participants were not informed about the SWAT and so were unaware the recruitment material they received had been chosen through random allocation.

### Statistical analysis

Analysis was conducted in STATA v18 [30]. Participants were included in the analyses in the groups to which they were allocated for the SWAT. All analysis models were interpreted with statistical significance at the 5% level.

Site characteristics and the flow of patients through the screening and recruitment process are presented by allocation and overall. Continuous data are summarised using descriptive statistics (n, mean, standard deviation, median, interquartile range, minimum and maximum), while categorical data are reported as counts and percentages.

The difference between recruitment rate in each SWAT group (primary outcome) and the difference between participants screened but not randomised (secondary outcome) was assessed using mixed effects logistic regression at the participant level, including the minimisation factors as fixed effects and site as a random effect. The difference between retention rate (secondary outcome) used the same logistic regression model as for the primary outcome, with the addition of the host trial allocation as a fixed effect.

We calculated the average cost per infographic as the sum of printing, preparation, and postage of the resources to sites. Staff time was calculated as the time spent undertaking SWAT activities (for example, filling and labelling envelopes) multiplied by their associated hourly pay rate, determined using the midpoint of the grade band (University of York) for each member of staff involved in SWAT. Postage costs were calculated using second class Royal Mail Mailmark franking rates. Cost per infographic was calculated as the total cost for each component, divided by the number of infographics. If the primary analyses identified higher recruitment in the SWAT intervention group (irrespective of statistical significance) the cost per additional participant would be calculated by dividing the total costs by the number of additional participants retained.

An exploratory meta-analysis was conducted, including the SWHSI-2 and BASIL + trials. The effect estimates of the intervention on recruitment rate were combined using a random effects meta-analysis model.

## Results

**Fig. 1:**
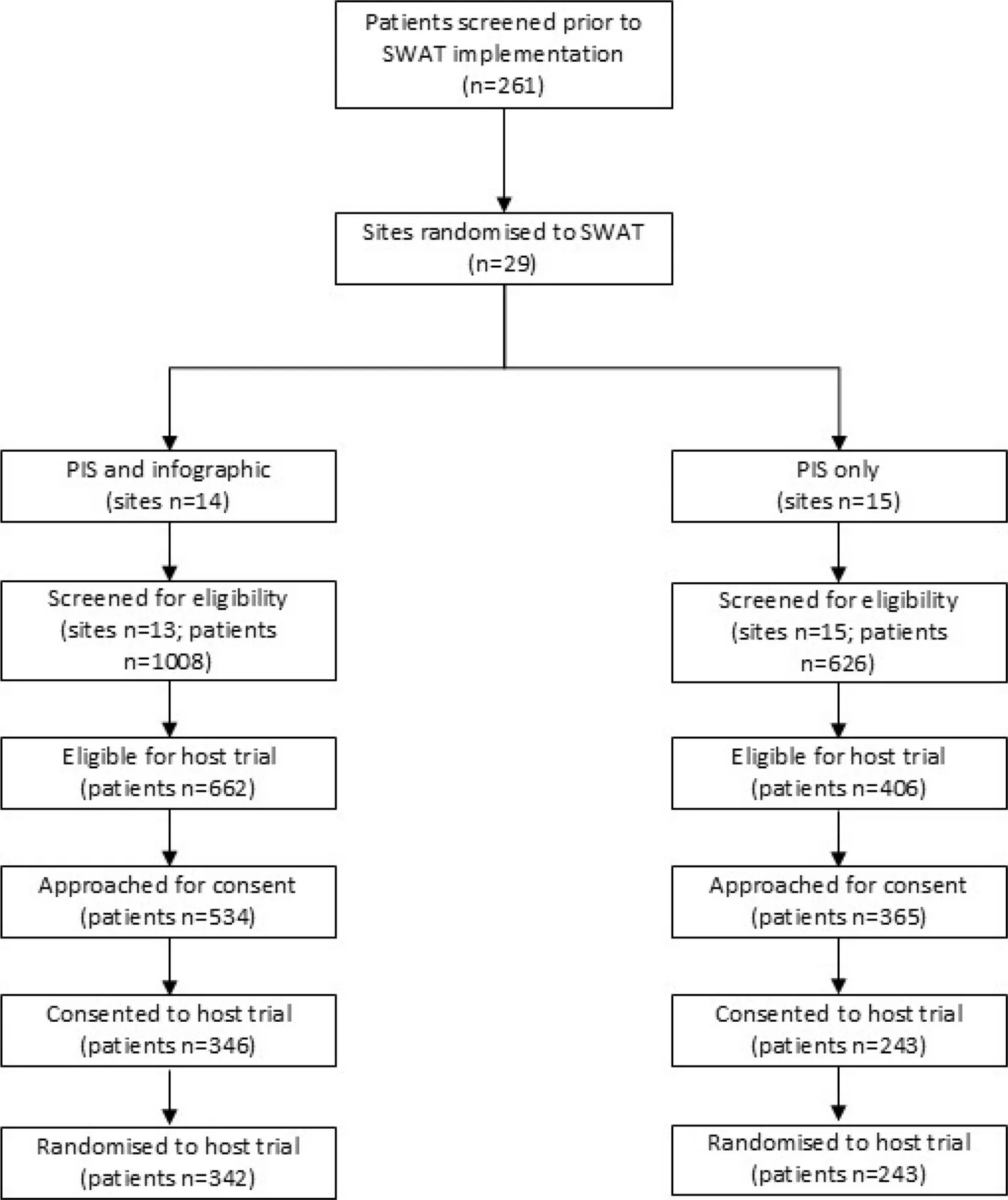
Study flow diagram

The SWAT ran between 09.03.2020 (SWAT implementation date) and 13.01.2023 (SWHSI-2 end of recruitment). Figure 1 outlines the flow of participants through the SWAT.

All 29 participating sites were randomised to the SWAT (14 intervention; 15 control). Table 1 describes site characteristics used for minimisation factors (expected site size and site surgical specialty). Some differences between the groups were noted. Sites allocated to the PIS plus infographic group tended to have fewer expected eligible participants per month than sites in the PIS only group (mean 8.2 vs 13.8) and were more likely to recruit cross specialty (71.4% vs 66.7%).

**Table 1:** Site baseline characteristics

Site characteristic	PIS only (*n* = 15)	PIS and infographic (*n* = 14)	Total (*n* = 29)
Expected number of eligible participants per month			
Mean (SD)	13.8 (24.4)	8.2 (7.9)	11.1 (18.3)
Median (Q1-Q3)	8 (3–11)	6 (2–10)	7 (3–10)
Min, max	2.0, 100.0	2.0, 30.0	2.0, 100.0
Expected number of eligible participants per month *N* (%)			
Above median (> 7)	8 (53.3)	6 (46.2)	14 (48.3)
Below median (≤ 7)	7 (46.7)	8 (57.1)	15 (51.7)
Site surgical specialty, *N* (%)			
Cross specialty	10 (66.7)	10 (71.4)	20 (69.0)
Single specialty	5 (33.3)	4 (28.6)	9 (31.0)

Patients were screened for inclusion in the SWHSI-2 trial by first assessing eligibility against the specified inclusion/exclusion criteria. Once eligibility was confirmed, patients were approached for consent and, if this was given, were randomised. From the SWAT implementation Date, 1634 screening forms were returned (PIS plus Infographic *n* = 1008 (61.7%); PIS only *n* = 626 (38.3%)). Of the 29 sites randomised, 28 sites screened at least one patient and were included in the primary analysis (one intervention site did not screen or recruit any patients). Table 2 displays the number of patients in each group and overall at different stages of the screening process.

**Table 2:** Patient screening figures by allocation and overall

	PIS plus infographic	PIS only	Total
Screened, *n*	1008	626	1634
Eligible, *n* (% of screened)	662 (65.7)	406 (64.9)	1068 (65.4)
Approached, *n* (% of eligible)	534 (80.7)	365 (89.9)	899 (84.2)
Consented, *n* (% of approached)	346 (64.8)	243 (66.6)	589 (65.5)
Randomised, *n* (% of consented; % of eligible)	342 (98.8; 51.7)	243 (100.0; 59.9)	585 (99.3; 54.8)

### Primary outcome—recruitment rate of eligible patients into the host trial

In total, 1068 patients were eligible to be consented and randomised to the host trial (PIS plus infographic *n* = 662 (62.0%); PIS only *n* = 406 (38.0%)). Of these, 585 patients were randomised into the SWHSI-2 trial (PIS plus infographic, 342/662 (51.7%); PIS only: 243/406 (59.9%)). There was no evidence of a difference in the likelihood of embedded trial participants being randomised into SWHSI-2 between the two groups (OR 0.61, 95% CI 0.23 to 1.62, *p* = 0.32; Table 3). The intra-cluster correlation coefficient (ICC) associated with site for the primary outcome was 0.29 (95% CI 0.15 to 0.47), the average cluster size was 38, and the coefficient of variation was 1.1.

**Table 3:** SWAT primary results

	PIS plus infographic	PIS only	*p*-value
Randomised to host trial, *n* (%)	342/662 (51.7%)	243/406 (59.9%)	0.32
Adjusted odds ratio (95% CI)	0.61 (0.23 to 1.62)		
Adjusted risk difference (95% CI)	−9.0% (−26.8% to 8.7%)		

Two post hoc sensitivity analyses were conducted as recommended by the peer-reviewer. The approach rate for consent among eligible patients was higher in the PIS only group (89.9%) than in the PIS plus infographic group (80.7%) and since this is an intermediary step between being eligible and randomised, it was thought this difference could bias the comparison of recruitment rates between the SWAT groups. The approach rate for each site was calculated as the proportion of participants approached out of those eligible, and this proportion was included as a site-level fixed effect in the primary analysis model. This reduced the magnitude of the difference to OR 0.93 (95% CI 0.43 to 2.02, *p* = 0.85). The ICC was also reduced to 0.18 (85% CI 0.08 to 0.35) which implies that the difference in approach rates was responsible for a proportion of the correlation of outcomes within sites. The difference in approach rates is largely driven by one intervention site with an approach rate of 35%, when the rate for all other sites is > 65%. Among the eligible participants not approached for consent at this site, the reason given was ‘patient to receive NPWT’ for half of them, largely due to clinician preference. This example demonstrates that clinical culture and recruitment practices differed between sites beyond just the use, or not, of the infographic.

When the number of clusters is small (typically < 30–40) [31], analyses of cluster trials can yield biased standard errors and inflated type I error rates without appropriate small-sample corrections. A small-sample correction was not initially applied to the primary analysis and so this was explored in a post-hoc sensitivity analysis. Two possible approaches to this for binary outcomes are to use a between-within correction in a generalized linear mixed model; or the Mancl and DeRouen correction in generalized estimating equations [32]. The latter was implemented in Stata and provided an OR of 0.75 (95% CI 0.31 to 1.84, *p* = 0.52). When additionally adjusting for approach rates, the difference becomes null (OR 1.03, 95% CI 0.52 to 2.04, *p* = 0.94).

### Secondary outcome—retention rate

From the SWAT implementation date, 585 participants were randomised into the host trial. At the 3-month follow-up, 367 (62.7%) participants returned the questionnaire; 343 (58.6%) participants returned the 6-month questionnaire; and 307 (52.5%) participants returned the 12-month questionnaire.

Return rates were higher in the PIS plus infographic group at each timepoint (Table 4); however, there was no statistically significant difference in the likelihood of embedded trial participants returning their follow-up questionnaires between the two groups at any timepoint: 3-months (OR 1.33, 95% CI 0.83 to 2.16, *p* = 0.24); 6-months (OR 1.26, 95% CI 0.78 to 2.02, *p* = 0.35); or 12-months (OR 1.36, 95% CI 0.97 to 1.90, *p* = 0.07).

**Table 4:** SWAT secondary results (retention rate)

	PIS plus infographic		PIS only	*p*-value
Month-3 follow-up				
Returned questionnaire, *n* (%)	221/342 (64.6%)		146/243 (60.1%)	0.24
Adjusted odds ratio (95% CI)	1.33 (0.83 to 2.16)			
Adjusted risk difference (95% CI)	6.4% (−4.1% to 17.0%)			
Month-6 follow-up				
Returned questionnaire, *n* (%)	204/342 (59.7%)	139/243 (57.2%)		0.35
Adjusted odds ratio (95% CI)	1.26 (0.78 to 2.02)			
Adjusted risk difference (95% CI)	5.3% (−5.7% to 16.2%)			
Month-12 follow-up				
Returned questionnaire, *n* (%)	191/342 (55.9%)	116/243 (47.7%)		0.07
Adjusted odds ratio (95% CI)	1.36 (0.97 to 1.90)			
Adjusted risk difference (95% CI)	7.6% (−0.7% to 15.9%)			

### Secondary outcome—proportion of patients screened that were not randomised to the host trial

Of the 1634 patients screened, 1049 (64.2%) patients were not randomised to the host trial (PIS plus infographic, 666/1008 (66.1%); PIS only, 383/626 (61.2%); OR 1.38, 95% CI 0.45 to 4.26, *p* = 0.57) (Table 5). Of those not randomised, 566 (54.0%) patients were ineligible, 169 (16.1%) were not approached under advice from the treating clinician, 310 (29.6%) patients did not consent, and 4 (0.4%) had consented but were not randomised.

**Table 5:** SWAT secondary analysis results–participants not randomised out of those screened

	PIS plus infographic	PIS only	*p*-value
Not randomised to host trial, *n* (%)	666/1008 (66.1%)	383/626 (61.2%)	0.57
Adjusted odds ratio (95% CI)	1.38 (0.45 to 4.26)		
Adjusted risk difference (95% CI)	5.8% (−14.1% to 25.6%)		

### Secondary outcome—cost per participant

The total cost of the SWAT was £357.26, which equates to £0.06 per infographic (Table 6). The costs of distributing these to sites were negligible (£0.29 per site) given that the infographics were distributed to sites along with other study supplies. As no statistically significant effect of the intervention was identified, a cost per additional participant was not calculated.

**Table 6:** Costs associated with the infographic SWAT

Task	Total cost	No infographics	Cost per infographic
Printing infographic*	£353.18	6000	£0.06
Packaging^+^ and postage^!^	£4.08	14 (sites)	£0.00 (cost per site £0.29)

*A total of 6000 infographics were ordered for the SWAT, sufficient to allow provision of an infographic to all eligible participants identified at each SWAT intervention site

^+^Packaging was completed by a University of York Grade 3 member of staff with the salary midpoint of the band used for calculations. Preparation and packaging took approximately 15 min (0.25 h) to complete at a rate of approximately 60 s per site randomised to the infographic

^!^Infographics were sent to trial sites, along with study case report forms and freepost envelopes as part of the study set up pack. Costs of delivery are based on a proportion (10%) of the cost of delivery as per University of York mailroom charges (£9.50)

### Harms

Data in relation to harms or unintended effects were not collected, nor were any noted during the conduct of this SWAT.

### Meta analysis

**Fig. 2:**
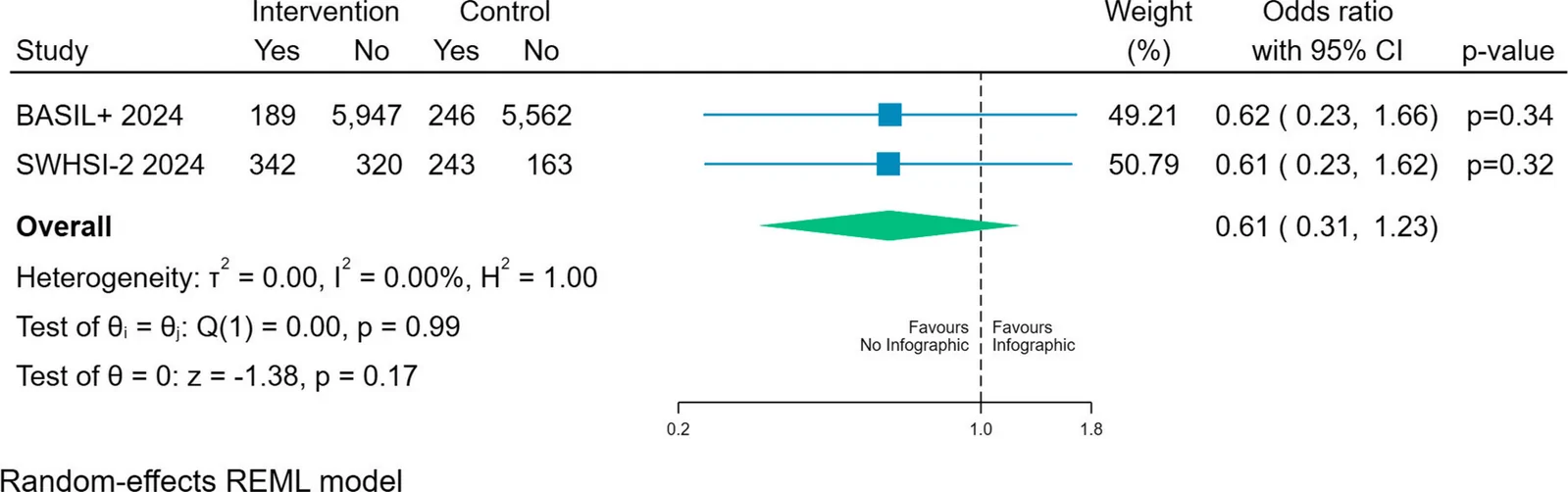
Meta-analysis of infographic SWAT for BASIL + and SWHSI-2 studies

A meta-analysis comparing rates of recruitment in the SWHSI-2 and BASIL + trials [23] was performed (Fig. 2). Overall, patients allocated to receive the infographic when approached were 0.61 times less likely to be recruited into the trial (OR = 0.61, 95% CI from 0.31 to 1.23). The I^2^ and τ^2^ statistics were both zero, indicating no heterogeneity.

## Discussion

This SWAT found no evidence of a difference in the likelihood of participants being randomised into the SWHSI-2 trial whether they were allocated to receive an infographic alongside their PIS or not. The effect estimate from this SWAT suggests that the intervention group (PIS plus infographic) was less likely to be recruited into the SWHSI-2 trial than the control group (PIS) (OR 0.61, 95% CI 0.23 to 1.62). However, there is significant uncertainty surrounding this estimate with confidence intervals suggesting that use of an infographic could either moderately reduce or increase recruitment. This finding is consistent with that of the BASIL + infographic SWAT which found that participants allocated to receive an infographic were less likely to be recruited [21]. Caution must therefore be taken in interpretation of the findings and their applicability to other trials given the uncertainty present.

Results were similar (BASIL + OR 0.62; SWHSI-2 OR 0.61); however, for both studies, there was limited certainty around this estimate with wide confidence intervals implying an imprecise estimate that is compatible with both a moderate reduction and a moderate increase in recruitment. In this SWAT, additional post hoc sensitivity analyses adjusting for differing approach rates among the sites and introducing a small-sample correction produced results that varied from the primary analysis implying the results are not very robust and must be interpreted with caution.

Considering the ICC, the average cluster size and the coefficient of variation, we estimate that the design effect for the primary analysis would be somewhere between 12 and 25 [33], which results in an approximate effective sample size of between 43 and 89 in an individually randomised trial. Assuming a recruitment rate of 60% in the control group, such small-sample sizes would have 80% power to detect only a very large absolute difference to 85–95%. Therefore, the trial was underpowered to detect the smaller differences observed.

The intervention was relatively low cost at £0.06 per infographic; however, the cost per additional participant retained was not calculated due to the absence of any evidence of effect.

Given the low cost of the intervention and the limited certainty around the primary outcome estimate, further replication of this SWAT is recommended to enable identification of a robust and definitive conclusion to the effectiveness of infographics as a RCT recruitment strategy.

### Strengths and limitations

A limitation of this SWAT was the method of intervention delivery. Recruiting sites randomised to the SWAT intervention were provided with copies of the infographic and were asked to utilise this when providing participant information to patients. As the intervention delivery was reliant on the recruiting study teams, in the context of busy clinics, wards, or visits, it is possible that some patients were not provided with the infographic leading to potential non-adherence. As data was not collected on whether an infographic was used with each patient at a SWAT intervention site compliance to the intervention was not assessed. While this information would not have influenced the results, since an intention-to-treat analysis approach was used, it would have provided useful context to assess causality. Further SWATs should therefore implement a robust method of monitoring infographic use to ensure that the true effect of the intervention is not inadvertently diluted. Randomisation at a site level was used to limit contamination; however, this may have instead resulted in differences in approaches employed by sites with regards screening, approach, and consent practices. For example, two sites allocated to the control group had 100% conversion rates from screening to randomised which contributes to the differential rates observed, compared to a 38% screening to randomisation conversion rate in the highest recruiting site allocated to the intervention.

This may also have been compounded given the intervention was implemented in SWHSI-2 on 09.03.2020, immediately prior to a study recruitment pause (with effect from 23.03.2020) due to COVID-19 restrictions. Between SWAT implementation and recruitment pause, only 14 participants were screened at 9 sites (7 intervention; 2 control) with only 10 participants who could potentially have received a PIS plus infographic (assuming the patient was eligible and approached for consent). Therefore, the SWAT was not fully embedded into the trial until individual sites recommenced recruitment following local governance review. Not all sites recommenced host trial and so SWAT activity at the same time which may have impacted both on numbers screened and approached and recommencement of intervention use.

It is not possible to compare participant-level characteristics for the SWAT groups as we did not collect demographic data at screening. Information on age, gender, ethnicity, etc. was collected at baseline (post consent, pre-randomisation) and so is known only for participants who were randomised to the SWHSI-2 trial. The SWHSI-2 host trial population was largely male (74.8%) and of white ethnicity (91.8%), with a median age of 63 years (IQR 55 to 72 years). Given these characteristics, it is not anticipated that proportions were substantially different for the SWAT population. The findings from this study are therefore generalisable only to this population with further replications in different populations remaining warranted.

### Implications for trial practice and SWAT research

Infographics as an adjunct to routinely provided participant information documents as a recruitment method requires further evaluation to confirm or refute intervention effectiveness. As a result, this intervention should currently only be used when being evaluated in a SWAT.

Given the results are only generalisable to the individual SWAT population, and as this is similar to the population of the earlier SWAT replication (older, white population) [21], sub-group analyses were not conducted. It is recommended that further replications be completed in additional populations, with a particular focus on younger and/or ethnically diverse populations, and that associated sub-group analyses are considered as part of future updates to the meta-analysis.

The associated meta-analysis suggests that patients not receiving an infographic were more likely to be recruited into both studies. This meta-analysis is small with only two studies, which means the between-study variance estimates are likely to be unstable; therefore, the findings should be interpreted with caution. The hope is that this meta-analysis will be added to in the future as more trials of an infographic are conducted. Additionally, the infographic used in BASIL + differed in content and style to that used in SWHSI-2 and so further exploration of infographic content and design is also warranted. Previously published research has indicated that approximately a quarter of potential trial participants provided consent without reading any of the information provided [34]. As a result, inclusion of a process evaluation to explore factors which influence patient engagement with research materials, how patients view and use infographic-format consent materials, the impacts of such materials on those with a range of health-literacy levels and across a range of cultures is recommended. This will facilitate understanding of the behavioural mechanisms integral to recruitment and support the further development of infographic and other information interventions, while ensuring that these are developed appropriately to facilitate equality, diversity and inclusivity.

## Conclusion

We conclude that given the relatively low statistical power of this second SWAT replication, the wide confidence intervals from the primary analysis, and varying findings from post-hoc sensitivity analyses, conclusions regarding a potential reduction in recruitment with use of the infographic should be interpreted cautiously. We intend that these findings should contribute to the accumulating SWAT evidence. Further replications are therefore required, ideally in younger and/or non-Caucasian populations, to distinguish conclusively whether an infographic offers an effective strategy for trials recruitment.

### Registration

Host Trial: ISRCTN18254597. Prospectively registered on 11th April 2017.

SWAT: MRC Hub for Trials Methodology Research SWAT repository #116 Registered 13.04.2020.

https://www.qub.ac.uk/sites/TheNorthernIrelandNetworkforTrialsMethodologyResearch/FileStore/Filetoupload,959361,en.pdf.

## Supplementary Information

Additional file 1: Supplement 1 SWHSI-2 infographic.

Additional file 2: Supplement 2 SWHSI-2 PIS.

Additional file 3.

## Data Availability

All data requests should be submitted to the corresponding author for consideration. Access to anonymised data may be granted following review.
