# Supplementary material for: SWAT 116: the effectiveness of an infographic to improve trial recruitment—a randomised study within a trial (SWAT)
Source: Trials. 2026 Jul 7;27:587. doi: 10.1186/s13063-026-09830-0 (PMC13621630; doi:10.1186/s13063-026-09830-0)
Supplement: Supplementary file 1 [file 13063_2026_9830_MOESM1_ESM.pdf]

# Would you like to take part?

## We are running a study to investigate which treatment heals surgical wounds better…

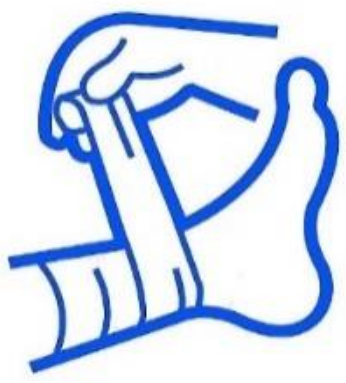

**Wound dressings - absorbing wound fluid and protecting the wound from bacteria**

**OR**

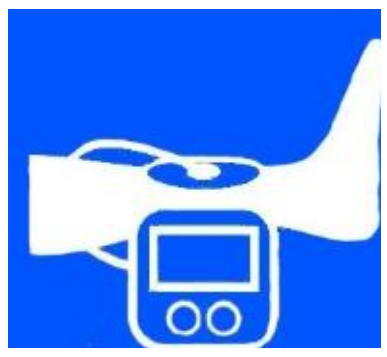

**Negative Pressure Wound Therapy (NPWT) - using a suction pump to remove wound fluid**

## Research with small numbers of people suggests that different treatments *may* …

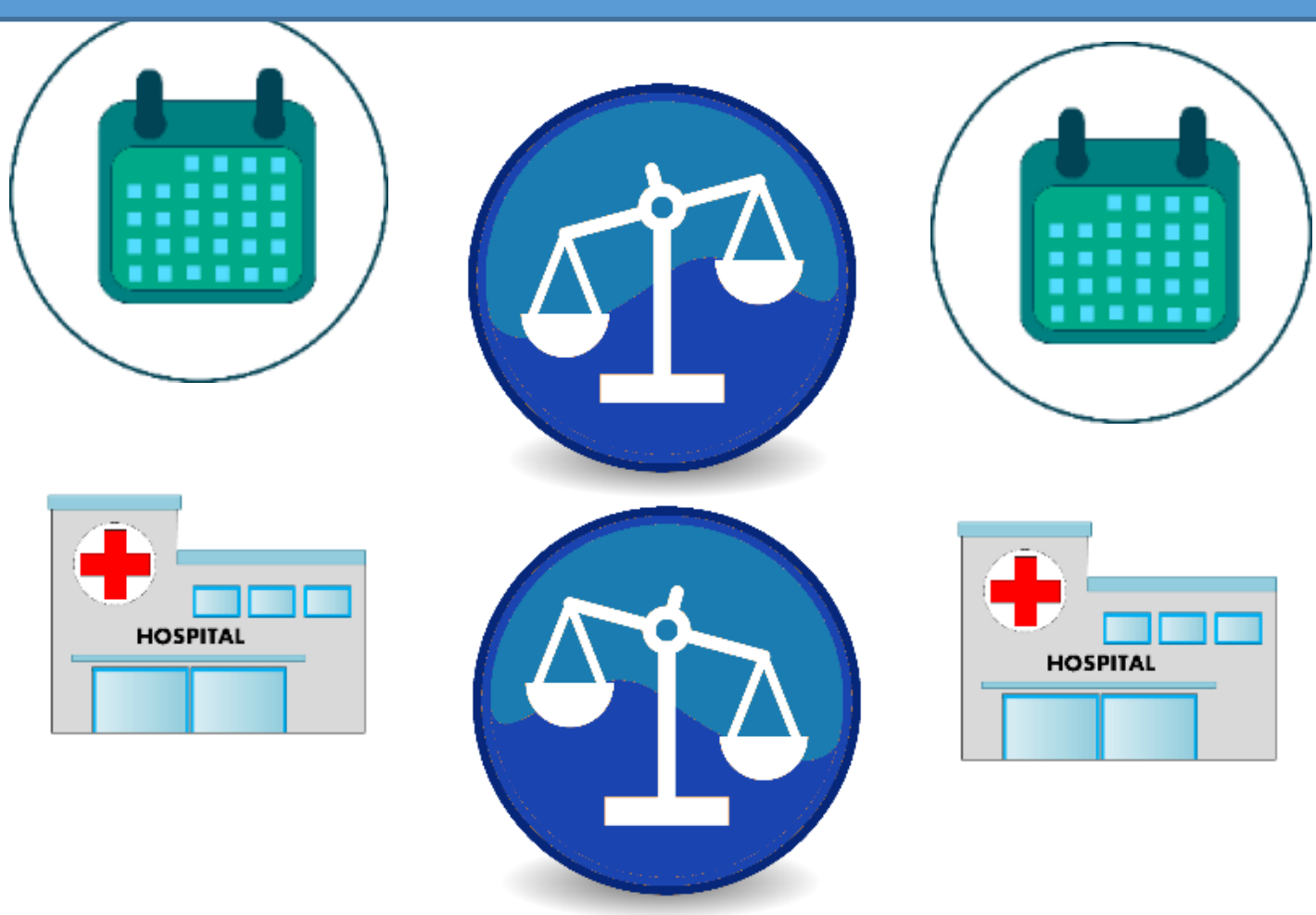


**Reduce the time it takes a wound to heal**

**Reduce the time someone spends in hospital**

## … but we do not know which treatment works best.

**Our study will compare these treatments and measure what happens to people during their care. In the future this information will help people make more informed treatment choices.**

FUNDED BY NIHR | National Institute for Health Research

This study is funded by the National Institute for Health Research (NIHR) Health Technology Assessment Programme (17/42/94). The views expressed are those of the author(s) and not necessarily those of the NIHR or the Department of Health and Social Care.
