# Supplementary material for: SWAT 116: the effectiveness of an infographic to improve trial recruitment—a randomised study within a trial (SWAT)
Source: Trials. 2026 Jul 7;27:587. doi: 10.1186/s13063-026-09830-0 (PMC13621630; doi:10.1186/s13063-026-09830-0)
Supplement: Supplementary file 2 [file 13063_2026_9830_MOESM2_ESM.pdf]

Surgical Wounds Healing by Secondary Intention (SWHSI-2) Trial - Can you help?

WE WOULD LIKE TO INVITE YOU TO TAKE PART IN A RESEARCH STUDY

Before you decide whether to take part, it is important for you to understand why the research is being done and what taking part will involve.

Please read the following information carefully and discuss this with anyone else you wish such as a friend, nurse, doctor or relative.

We are very happy to provide more information if anything is unclear and can be contacted using the details at the end of the leaflet.

Please take as much time as you need to decide whether you want to take part.

THE SWHSI-2 TRIAL IN BRIEF

We are running this study to find out how best to treat surgical wounds which are left to heal from the bottom up.

We want to find out if a type of dressing where a vacuum pump is attached to the wound (negative pressure wound therapy) is better, worse or the same as using normal dressings without this pump. Currently, there is not enough evidence available to answer this question.

In the study some people will receive negative pressure wound therapy, whilst others will receive dressings without the pump. To make this study highly reliable, the treatment people receive must be selected by chance. Every patient will still receive the best care available – we are only measuring the difference (if any) that one treatment may have against the other.

If you take part, over the next year, we will ask you to complete some questionnaires either by telephone or by post (we will provide a freepost envelope for you to return these to us). We will also ask a nurse to contact you by telephone on a weekly basis to check how your wound is healing. A photograph of your wound will be taken at the start of the study and again once it has healed. We will also ask you to take a photo of your healed wound if you feel comfortable to do so.

The study is being run across the United Kingdom and <<INSERT SITE NAME>> are running this study in your area.

The study is being run by the Universities of Hull and York and Hull University Teaching Hospitals NHS Trust.

INSIDE THIS LEAFLET

Page 2

- What is the purpose of this study?
- Why have I been chosen?
- Do I have to take part?
- What will happen if I take part?

Page 3

- What treatment is being tested?
- What are the benefits of taking part?
- What are the disadvantages and risks of taking part?
- What happens if something goes wrong?
- What if new information becomes available?

Page 4

- What happens when the research stops?
- What happens if I no longer wish to take part in the study?
- Will taking part in this study be kept confidential?
- What will happen to the results of the study?

Page 5

- Who is organising and funding the research?
- Who has reviewed the study?
- How have patients and the public helped to design the study?
- What do I do now?
- Contact details

This project was funded by the National Institute for Health Research Health Technology Assessment Programme (project number 17/42/94). The views expressed are those of the authors and not necessarily those of the NHS, the NIHR or the Department of Health and Social Care.

SWHSI-2

York Trials Unit

WHY ARE WE DOING THIS STUDY?

After an operation, many wounds are closed by the surgeon using stitches or staples to help them heal. However, some surgical wounds cannot be closed this way and are left open to heal 'from the bottom up'. Alternatively, a closed wound may open up again on its own and so is then left open to heal. These types of open wounds are also called "surgical wounds healing by secondary intention" (SWHSI).

The normal treatment for these wounds is wound dressings. A vacuum pump, called "negative pressure wound therapy" is sometimes also used to apply suction to the wound.

We are conducting this study because currently both of these treatments are used regularly in the NHS, however we are unsure whether one is more effective at healing wounds than the other.

To find the answer to this question, the SWHSI-2 Trial will recruit and gather important information from a large number of patients with an open surgical wound.

WHY HAVE I BEEN ASKED TO TAKE PART?

Your nurse and / or doctor have decided that the type of wound you have would be equally suitable for either of the two different treatments in this study. Information about how your wound and treatment progresses, is important to include in this study.

DO I HAVE TO TAKE PART?

No, taking part is completely voluntary. It is entirely up to you whether or not to be involved, and you are free to change your mind at any time.

If you do decide to take part you will be given this information sheet to keep and you will be asked to sign a consent form to confirm your decision.

WHAT WILL HAPPEN IF I TAKE PART?

Provided there are no health circumstances which would make it inappropriate to include you in this study, and you are happy to take part, we will ask you to complete a study consent form. You will be given a copy to keep and a copy will be sent to York Trials Unit.

We will then assess and photograph your wound, and ask you some questions about it and your general health.

You will then be allocated to one of two groups: one group will have treatment with negative pressure wound therapy and one will receive other dressings.

Everyone taking part is allocated to a group at random so you will have an equal chance of receiving either treatment, in the same way that tossing a coin gives an equal chance of getting 'heads' or 'tails'. The researchers will not know which group you will be in until after you agree to take part. There is no reason to feel disadvantaged or disappointed by which treatment you are allocated to. Both are currently used regularly and you will always receive the very best care and treatment we can provide. We are running this study because at present there is not enough information to show that either treatment is any better than the other.

You will continue seeing your health professional for your wound treatments to be carried out as normal.

A member of the research team will contact you each week by telephone to check how your wound is healing. You will also be posted a short questionnaire booklet to complete, asking about your health and your wound, at 3 months, 6 months and 12 months from joining the study. As a small thank you gesture for your help, we will send you £5 at the 6 and 12 month follow up visits. Your wound will be photographed by the research team on the first week after it has healed, we may also ask you if you can take a photo yourself. You may need to attend the hospital again for this visit. If this is required we will cover your travel expenses for attending the visit. We will also contact you by telephone for two further weeks, to check your wound remains healed.

<u>Future research</u>

The information collected may be used to support other research in the future. Any information shared will be done so anonymously.

WHAT TREATMENT IS BEING TESTED?

Please be assured, there are **no** experimental or unusual treatments involved in this study. Both negative pressure wound therapy and wound dressings are routinely used to treat open surgical wounds in the NHS. Your treatment will be no different to any of these current methods.

Negative pressure wound therapy involves the application of a pump which provides a suction force (negative pressure) across a wound surface.

Wound dressings used are designed to absorb any wound fluid and to protect the wound from bacteria.

Additional information about both of these treatments will be provided to you by your nurse or doctor.

Neither treatment should be uncomfortable; you will be monitored regularly to check how you are finding the treatment. This is what would happen normally with both treatments in the NHS.

WHAT ARE THE BENEFITS OF TAKING PART?

Every effort will be made to help your wound improve with either treatment in the usual way. However, we cannot say whether your wound will improve more quickly by your being in the study. The information we get from this study may help us to treat people with similar open wounds more effectively in the future.

WHAT ARE THE RISKS OF TAKING PART?

Being in this study will not harm or disadvantage your care in any way. Side effects in both treatments are very uncommon.

Negative Pressure Wound Therapy

Negative pressure wound therapy machines may present a trip hazard for you or your family. Care should be taken when moving around.

If you have a stomach wound, you may be at increased risk of developing a fistula, an abnormal opening between organs and the skin. You will be monitored regularly as part of usual NHS care.

Wound Dressings

You may have to undergo frequent dressing changes, sometimes daily, which will usually be completed by your nurse.

WHAT HAPPENS IF SOMETHING GOES WRONG?

If you have a concern about any aspect of the study, you should speak to your research doctor or nurse who will do their best to answer your queries. If you remain unhappy and wish to complain formally, you can obtain advice from your local Patient Advice and Liaison Service. Contact details are provided at the end of this leaflet.

In the unlikely event that this should occur, normal NHS negligence procedures apply. There is no special compensation system involved for the study. If you are harmed due to someone's negligence, then you should seek independent advice about any legal action. If you wish to complain, or have any concerns about any aspect of the way you have been approached or treated during the course of this study, the normal NHS complaints services are available to you.

In an emergency you should contact your research doctor or nurse. The name of a contact nurse and telephone number where they can be reached is provided at the end of this leaflet.

WHAT IF NEW INFORMATION BECOMES AVAILABLE?

Sometimes during a trial, new information regarding the treatments being studied becomes available.

If this happens, your nurse or doctor will tell you about it and discuss with you if you want to continue in the study, and if it is appropriate for you to continue to do so.

If you decide to withdraw from the study your care will continue in line with routine practice.

If you decide to continue, then you will be asked to sign an updated consent form.

WHAT HAPPENS WHEN THE RESEARCH STOPS?

When the study ends, you will return to standard NHS care either with your GP or consultant

If your wound does not fully heal during the study, your care will continue with the treatment your doctor or nurse feels is best for your wound.

WHAT HAPPENS IF I NO LONGER WISH TO TAKE PART?

If you agree to take part but later change your mind you can withdraw from the study at any time without giving a reason and your future care and treatment will not be affected.

Your usual rights to access, change or move your information are altered slightly as we need to manage your information in specific ways to ensure the research is reliable and accurate.

If you leave the study, we would still like to keep, and use the information we have collected from you as this is valuable to the study. If you do not wish us to keep or use this information please let the study team know. If you do allow us to use the information we have collected, to safeguard your rights, we will use the minimum personally identifiable information possible.

WILL TAKING PART IN THIS STUDY BE KEPT CONFIDENTIAL?

If you agree to take part we will only inform your GP and any doctor or nurse who may be treating you.

Information that is collected about you during the course of the research, and from your health records, will be held securely on paper or electronically at the study site and York Trials Unit, the centre organising the research. Information will be kept strictly confidential and will be held in line with the Data Protection Act (2018).

Your name, address and contact telephone number will be stored securely at the University of York to allow us to contact you about study questionnaires, to make sure that relevant information about the study is recorded for your care, and to oversee study quality. Your details will not be passed to anyone outside of the research team.

Digital photographs of your wound, taken during the study will be transferred to York Trials Unit and stored securely. The images will be as anonymised as possible and your name will not be included in the image. If however, you have any identifying marks located close to your wound (e.g. a tattoo, a mole or scar) these may remain visible in the image.

Study information and your medical records may be viewed by individuals from the study team, the Sponsor, the Research Ethics Committee, the NHS Trust or UK regulatory authorities. These organisations may do this to check that the study is carried out to the highest standards.

The people who analyse the information will not be able to identify you and will not be able to find out your name, or contact details.

You will be given a participant ID number, which will be used as a code to identify you on all trial forms, photographs and questionnaires. These will be sent and stored securely and will not include your name or any details about you on them. Your name will only appear on your consent form, which will be sent separately to any trial data collected.

Anonymised results from the study may be stored indefinitely for other health and care research in the future. Any identifying information will be kept strictly confidential, and access will be limited to the original study and database teams, and this will not be used to contact you, to affect your care or to make future decisions about services available to you. Anyone looking at your records will have a duty of confidentiality to you as a research participant.

WHAT WILL HAPPEN TO THE RESULTS OF THE STUDY?

The results of the study will be made available to you once the trial has finished

The results of the study will be published in medical and nursing journals. You will not be identified in any publication or presentation arising from this study.

WHO IS ORGANISING AND FUNDING THE RESEARCH?

The study is funded by the National Institute for Health Research Health Technology Assessment Programme (Reference: 17/42/94). Your nurse and doctor are not receiving any money for conducting this research.

The study is being coordinated by the University of York – York Trials Unit. The study is sponsored by the Hull University Teaching Hospitals NHS Trust.

We will be using information from you and/or your medical records in order to undertake this research and the sponsor will act as the data controller for this study. This means that we are responsible for looking after your information and using it properly. Other collaborating institutions will also be processing your data and will keep identifiable information about you. You can find out more about how we use your information at: [INSERT SPONSOR LINK]

If you agree to take part in the trial, the University of York, and participating hospitals will keep information on you for a minimum of 5 years. Confidential destruction will then be arranged.

WHO HAS REVIEWED THE STUDY?

All research in the NHS is looked at by an independent group of people called a research ethics committee. The [INSERT NAME] Research Ethics Committee have reviewed and approved this study.

HOW HAVE PATIENTS AND THE PUBLIC HELPED TO DESIGN THE STUDY?

Patients have confirmed the importance of this research question and have reviewed the participant information sheet and other study documentation.

WHAT DO I DO NOW?

Having read this information you may decide:

Yes, I would like to take part

If you are interested in taking part please discuss this study with your doctor or nurse. If you decide to take part you will be given a copy of this information sheet to keep and will be asked to sign a consent form to confirm your decision.

No, I do not wish to take part

If you do not wish to take part you do not need to do anything. Thank you for reading this information.

I am unsure and would like more information

If you do not understand anything on this information sheet or would like further information please contact the nurse on the telephone number provided.

You are encouraged to ask questions if you wish before, during and after your treatment. If you have any questions, please contact the study contacts below:

Research Nurse: [INSERT SITE CONTACT DETAILS – INCLUDING OUT OF HOURS]

Alternatively you can contact:

Study Coordinator: [INSERT CONTACT DETAILS]

If you would like to talk to someone else for general information and advice on taking part in research please contact:

[INSERT LOCAL PALS DETAILS]
