# Supplementary material for: SWAT 116: the effectiveness of an infographic to improve trial recruitment—a randomised study within a trial (SWAT)
Source: Trials. 2026 Jul 7;27:587. doi: 10.1186/s13063-026-09830-0 (PMC13621630; doi:10.1186/s13063-026-09830-0)
Supplement: Supplementary file 3 [file 13063_2026_9830_MOESM3_ESM.docx]

**Trial Forge Guidance 4: A guideline for reporting the results of randomised Studies Within A Trial (SWATs)**

|  | |  | **Page Number** |
| --- | --- | --- | --- |
| **Title and Abstract** | | |  |
| **1a** | The term ‘SWAT’ should be used in the title & SWAT registry number should be included if available | | Page 1 |
| **1b** | Structured summary: Background, Methods, Results, Conclusion  Details of the host trial(s) included in which the SWAT intervention was evaluated | | Page 2 |
| **1c** | Keywords Including: ‘SWAT’; ‘Study Within A Trial’; the trial process targeted (e.g., ‘recruitment methods’)]; embedded randomised controlled trial | | Page 2 |
| **Introduction; Background and objectives** | | |  |
| **2a** | Scientific background and explanation of rationale for the SWAT If a replication SWAT cite previous SWAT evaluations undertaken as part of the rationale | | Page 3 |
| **2b** | Specific objectives or hypotheses for the SWAT | | Page 4 |
| **Methods; Trial design** | | |  |
| **3a** | - Description of the SWAT (such as parallel, factorial, cluster), including allocation ratio, SWAT registration, details of host trials in PICO format and ethical approval | | Page 4 |
| **3b** | State changes (with reasons) to methods of SWAT following commencement | | Page 4 |
| **Participants** | | |  |
| **4a** | State eligibility criteria in SWAT, including differences to those from the host trial(s) | | Page 5 |
| **4b** | Include setting(s) and location(s) where SWAT data was collected | | Page 5 |
| **Interventions** | | |  |
| **5** | Describe SWAT intervention to enable replication, including how and when interventions were administered and recruitment dates. Reference to the SWAT protocol for further details is acceptable if the protocol is available to the reader | | Page 5 |
| **Outcomes** | | |  |
| **6a** | State primary and secondary outcome measures for the SWAT. Include how and when they were assessed | | Page 5 |
| **6b** | Include changes (and reasons) to SWAT outcomes after commencement | | Page 5 |
| **Sample size** | | |  |
| **7a** | How sample size was determined for the SWAT.  SWATs are often individually underpowered due to the sample size being constrained by the host trial(s). A robust estimate of the effect of the SWAT intervention might therefore depend on the aggregation of replicated SWAT evaluations. | | Page 6 |
| **7b** | When applicable, explanation of any interim analyses and stopping rules for the SWAT | | Page 6 |
| **Randomisation:  Sequence generation** | | |  |
| **8a** | The method used to generate the random allocation sequence for the SWAT | | Page 6 |
| **8b** | Type of randomisation; details of any restriction (such as blocking and block size) | | Page 6 |
| **Allocation concealment mechanism** | | |  |
| **9** | The mechanism used to implement the random allocation sequence (such as sequentially numbered containers), describing any steps taken to conceal the sequence until interventions were assigned for the SWAT | | Page 6 |
| **Implementation** | | |  |
| **10** | Who generated the random allocation sequence, who enrolled participants, and who assigned participants to interventions for the SWAT | | Page 6 |
| **Blinding** | | |  |
| **11a** | If done, who was blinded after assignment to the SWAT interventions (for example, participants, care providers, those assessing outcomes), and how | | Page 6 |
| **11b** | If relevant, a description of the similarity of the SWAT interventions | | Page 6 |
| **Statistical methods** | | |  |
| 12a | Statistical methods used to compare groups for primary and secondary outcomes for the SWAT | | Page 6 - 7 |
| 12b | Methods for additional analyses, such as subgroup analyses and adjusted analyses | | Page 6 - 7 |
| **Results** | | |  |
| **Participant flow** | | |  |
| 13a | For each group, the numbers of participants who were randomly assigned, received intended SWAT intervention, and were analysed for the primary outcome of the SWAT | | Page 8 |
| 13b | For each group participating in the SWAT, losses, and exclusions after randomisation, together with reasons | | Page 8 |
| **Recruitment** | | |  |
| 14a | Dates defining the periods of recruitment and follow-up of the SWAT | | Page 8 |
| 14b | Why the SWAT ended or was stopped | | Page 8 |
| **Baseline data** | | |  |
| **15** | A table showing baseline demographic and clinical characteristics for each group. At a minimum, age, gender and ethnicity should be reported per group in addition to any demographics deemed relevant by the host trial team | | Page 9 |
| **Numbers analysed** | | |  |
| 16 | For each group of the SWAT, number of participants (denominator) included in each analysis and whether the analysis was by original assigned groups | | Page 10 - 13 |
| **Outcomes and estimation** | | |  |
| 17a | For each primary and secondary outcome, results for each group, and the estimated effect size and its precision (such as 95% confidence interval) | | Page 10 – 13 |
| 17b | For binary outcomes, the presentation of both absolute and relative effect sizes is recommended | | Page 10 – 13 |
| 17c | Costs associated with the SWAT | | Page 13 |
| **Ancillary analyses** | | |  |
| 18 | Results of any other analyses performed on the SWAT data, including subgroup analyses and adjusted analyses, distinguishing pre-specified from exploratory | | Page 10 – 13 |
| **Harms** | | |  |
| 19 | All important harms or unintended effects in each group that took part in the SWAT (for specific guidance, see CONSORT for harms) | | Page 13 |
| **Discussion** | | |  |
| 20 | Interpretation consistent with results, balancing benefits, and harms, and considering other relevant evidence | | Page 14-16 |
| **Limitations** | | |  |
| 21 | SWAT limitations, addressing sources of potential bias, imprecision, and, if relevant, multiplicity of analyses for the SWAT | | Page 15 |
| **Generalisability** | | |  |
| 22 | Generalisability (external validity, applicability) of the SWAT findings | | Page 14-16 |
| **Implications** | | |  |
| **23** | Implications for trial practice and SWAT research | | Page 15-16 |
| **Other information** | | |  |
| 24 | Registration  Registration number and name of trial registry | | Page 16 |
